# Tris(3,5-dimethyl-1*H*-pyrazole-κ*N* ^2^)(pyridine-2,6-dicarboxyl­ato-κ^3^ *O* ^2^,*N*,*O* ^6^)cobalt(II) monohydrate

**DOI:** 10.1107/S1600536809010538

**Published:** 2009-03-28

**Authors:** Kun-Hua Lin, Zhe-Yin Yu, Yan-Hua Zhong, Min Shao

**Affiliations:** a Department of Chemistry, College of Science, Shanghai University, Shanghai 200444, People’s Republic of China; b Instrumental Analysis and Research Center, Shanghai University, Shanghai 200444, People’s Republic of China

## Abstract

The reaction of Co(NO_3_)_2_·3H_2_O with pyridine-2,6-dicarboxylic acid and 3,5-dimethyl-1*H*-pyrazole in a 1:1:3 molar ratio affords the title complex, [Co(C_7_H_3_NO_4_)(C_5_H_8_N_2_)_3_]·H_2_O. The Co^II^ atom is coordinated by one pyridine-2,6-dicarboxyl­ate chelating ligand and three 3,5-dimethyl-1*H*-pyrazole ligands in a distorted octa­hedral geometry. Hydrogen-bonding interactions involving the coordinated carboxylate group, 3,5-dimethyl-1*H*-pyrazole and water help to consolidate the crystal structure

## Related literature

For the use of complexes with pyrazole-based ligands in studying the relationship between the structure and the activity of the active site of metalloproteins, see: Haanstra *et al.* (1990 ▶). For the coordination modes of pyrazole complexes, see: Grotjahn *et al.* (2003 ▶).
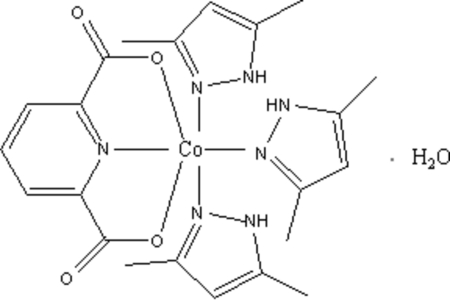


## Experimental

### 

#### Crystal data

[Co(C_7_H_3_NO_4_)(C_5_H_8_N_2_)_3_]·H_2_O
*M* *_r_* = 530.45
Triclinic, 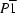

*a* = 8.4220 (8) Å
*b* = 11.9936 (12) Å
*c* = 13.1418 (13) Å
α = 75.1290 (10)°
β = 84.7720 (10)°
γ = 70.0940 (10)°
*V* = 1206.3 (2) Å^3^
*Z* = 2
Mo *K*α radiation
μ = 0.76 mm^−1^
*T* = 296 K
0.30 × 0.30 × 0.25 mm

#### Data collection

Bruker SMART CCD area-detector diffractometer
Absorption correction: multi-scan (*SADABS*; Sheldrick, 1996 ▶) *T*_min_ = 0.804, *T*_max_ = 0.833
6298 measured reflections
4183 independent reflections
3769 reflections with *I* > 2σ(*I*)
*R*_int_ = 0.014

#### Refinement

*R*[*F* ^2^ > 2σ(*F* ^2^)] = 0.030
*wR*(*F* ^2^) = 0.077
*S* = 1.03
4183 reflections
331 parameters
2 restraints
H atoms treated by a mixture of independent and constrained refinement
Δρ_max_ = 0.25 e Å^−3^
Δρ_min_ = −0.25 e Å^−3^

### 

Data collection: *SMART* (Bruker, 2000 ▶); cell refinement: *SAINT* (Bruker, 2000 ▶); data reduction: *SAINT*; program(s) used to solve structure: *SHELXS97* (Sheldrick, 2008 ▶); program(s) used to refine structure: *SHELXL97* (Sheldrick, 2008 ▶); molecular graphics: *SHELXTL* (Sheldrick, 2008 ▶); software used to prepare material for publication: *SHELXTL*.

## Supplementary Material

Crystal structure: contains datablocks global, I. DOI: 10.1107/S1600536809010538/bq2127sup1.cif

Structure factors: contains datablocks I. DOI: 10.1107/S1600536809010538/bq2127Isup2.hkl

Additional supplementary materials:  crystallographic information; 3D view; checkCIF report

## Figures and Tables

**Table 1:** Selected geometric parameters (Å, °)

Co1—N1	2.0407 (16)
Co1—N4	2.0798 (16)
Co1—O1	2.1453 (14)
Co1—O3	2.1522 (14)
Co1—N2	2.2336 (17)
Co1—N6	2.2477 (17)

**Table 2:** Hydrogen-bond geometry (Å, °)

*D*—H⋯*A*	*D*—H	H⋯*A*	*D*⋯*A*	*D*—H⋯*A*
N3—H3*A*⋯O1*W*^i^	0.86	2.22	2.926 (2)	139
N3—H3*A*⋯O1	0.86	2.61	3.048 (2)	113
N5—H5⋯O2^ii^	0.86	2.10	2.945 (2)	168
N7—H7⋯O2^ii^	0.86	2.08	2.838 (2)	146
N7—H7⋯O3	0.86	2.42	2.906 (2)	116
O1*W*—H1*WA*⋯O4^iii^	0.844 (17)	1.967 (18)	2.797 (2)	168 (3)
O1*W*—H1*WB*⋯O3^iv^	0.828 (17)	2.204 (19)	3.009 (2)	164 (3)

Symmetry codes: (i) 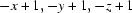; (ii) 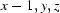; (iii) 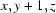; (iv) 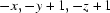.

**Fig. 1.**
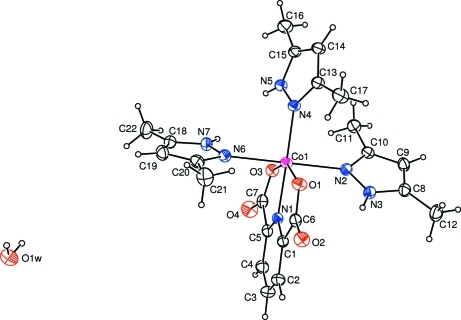
Molecular structure of (I) with displacement ellipsoids drawn at the 30% probability level.

**Fig. 2.**
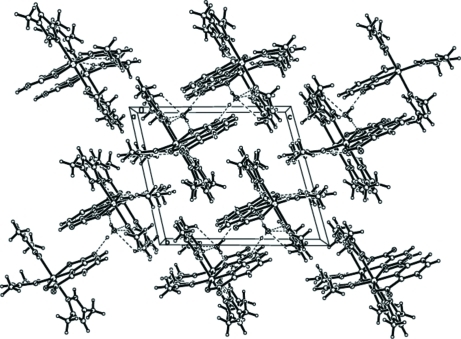
A packing diagram of (I).

## supplementary crystallographic information

### Comment

Complexes with pyrazole-based ligands are a frequent subject of chemical investigations giving an opportunity for a better understanding of the relationship between the structure and the activity of the active site of metalloproteins (Haanstra *et al.*, 1990). Nowadays, attention is paid to the design of various pyrazole ligands, and some coordination modes of pyrazole complexes were reported (Grotjahn *et al.*, 2003). In our systematic studies on transition metal comlexes with the pyrazole derivatives, the title compound was prepared and its X-ray structure is presented here.

The molecular structure of the title compound is shown in Fig. 1. The compound assumes a distorted octahedron geometry, formed by three 3,5-Dimethyl-1-*H*-pyrazole molecules and a pyridine-2,6-dicarboxylate. Tridentate ligand pyridine-2,6-dicarboxylate dianion chelates to the Co atom by a N atom of pyridine ring and two O atoms of carboxyl groups with a meridional configuration. Monodentate ligand 3,5-Dimethyl-1-*H*-pyrazole coordinated to the Co atom by N atoms of pyrazole rings. The bond distances of Co1—N1 and Co1—N4 are 2.0407 (16)Å and 2.0798 (16)Å (Table 1), which are shorter than the the bond distances of Co1—N2 and Co1—N6 with 2.2336 (17)Å and 2.2477 (17)Å.

### Experimental

An ethanol solution (6 ml) containing 3,5-Dimethyl-1-*H*-pyrazole(0.1153 g, 1.2 mmol) and Co(NO_3_)_2_.3H_2_O(0.0870 g, 0.3 mmol) was mixed with an aqueous solution (6 ml) of pyridine-2,6-dicarboxylic acid(0.0501 g, 0.3 mmol) and NaOH (0.0240 g, 0.6 mmol). The mixture was refluxed for 6 h. The solution was filtered after cooling to room temperature. Pink single crystals suitable for X-ray diffraction were obtained from the filtrate after 11 d.

### Refinement

The H atoms of water molecule were located in a difference Fourier map and refined freely. Methyl H atoms were placed in caculated positions with C—H distances = 0.96 Å and *U*_iso_(H) = 1.5*U*_eq_(C). Other H atoms were placed in caculated positions with C—H distances = 0.93 Å and N—H distances = 0.86 Å, and *U*_iso_(H) = 1.5*U*_eq_(C,*N*).

### Crystal data

**Table:**

[Co(C_7_H_3_NO_4_)(C_5_H_8_N_2_)_3_]·H_2_O	*Z* = 2
*M**_r_* = 530.45	*F*(000) = 554
Triclinic, *P*1	*D*_x_ = 1.460 Mg m^−^^3^
Hall symbol: -P 1	Mo *K*α radiation, λ = 0.71073 Å
*a* = 8.4220 (8) Å	Cell parameters from 4060 reflections
*b* = 11.9936 (12) Å	θ = 2.7–27.5°
*c* = 13.1418 (13) Å	µ = 0.76 mm^−^^1^
α = 75.129 (1)°	*T* = 296 K
β = 84.772 (1)°	Block, pink
γ = 70.094 (1)°	0.30 × 0.30 × 0.25 mm
*V* = 1206.3 (2) Å^3^	

### Data collection

**Table:**

Bruker SMART CCD area-detector diffractometer	4183 independent reflections
Radiation source: fine-focus sealed tube	3769 reflections with *I* > 2σ(*I*)
graphite	*R*_int_ = 0.014
φ and ω scans	θ_max_ = 25.1°, θ_min_ = 2.1°
Absorption correction: multi-scan (*SADABS*; Sheldrick, 1996)	*h* = −10→9
*T*_min_ = 0.804, *T*_max_ = 0.833	*k* = −14→14
6298 measured reflections	*l* = −13→15

### Refinement

**Table:**

Refinement on *F*^2^	Secondary atom site location: difference Fourier map
Least-squares matrix: full	Hydrogen site location: inferred from neighbouring sites
*R*[*F*^2^ > 2σ(*F*^2^)] = 0.030	H atoms treated by a mixture of independent and constrained refinement
*wR*(*F*^2^) = 0.077	*w* = 1/[σ^2^(*F*_o_^2^) + (0.0334*P*)^2^ + 0.7243*P*] where *P* = (*F*_o_^2^ + 2*F*_c_^2^)/3
*S* = 1.03	(Δ/σ)_max_ = 0.002
4183 reflections	Δρ_max_ = 0.25 e Å^−^^3^
331 parameters	Δρ_min_ = −0.24 e Å^−^^3^
2 restraints	Extinction correction: *SHELXL97* (Sheldrick, 2008), Fc^*^=kFc[1+0.001xFc^2^λ^3^/sin(2θ)]^-1/4^
Primary atom site location: structure-invariant direct methods	Extinction coefficient: 0.0176 (11)

### Special details

**Table:**

Geometry. All e.s.d.'s (except the e.s.d. in the dihedral angle between two l.s. planes)are estimated using the full covariance matrix. The cell e.s.d.'s are takeninto account individually in the estimation of e.s.d.'s in distances, anglesand torsion angles; correlations between e.s.d.'s in cell parameters are onlyused when they are defined by crystal symmetry. An approximate (isotropic)treatment of cell e.s.d.'s is used for estimating e.s.d.'s involving l.s. planes.
Refinement. Refinement of *F*^2^ against ALL reflections. The weighted *R*-factor *wR* andgoodness of fit *S* are based on *F*^2^, conventional *R*-factors *R* are basedon *F*, with *F* set to zero for negative *F*^2^. The threshold expression of*F*^2^ > σ(*F*^2^) is used only for calculating *R*-factors(gt) *etc*. and isnot relevant to the choice of reflections for refinement. *R*-factors basedon *F*^2^ are statistically about twice as large as those based on *F*, and *R*-factors based on ALL data will be even larger.

### Fractional atomic coordinates and isotropic or equivalent isotropic displacement parameters (Å^2^)

**Table:**

	*x*	*y*	*z*	*U*_iso_*/*U*_eq_	
Co1	0.33263 (3)	0.28250 (2)	0.246751 (19)	0.02818 (10)	
C1	0.5981 (2)	0.24913 (18)	0.39330 (15)	0.0295 (4)	
C2	0.6852 (3)	0.2085 (2)	0.48700 (17)	0.0392 (5)	
H2	0.7859	0.2228	0.4914	0.047*	
C3	0.6195 (3)	0.1461 (2)	0.57368 (17)	0.0457 (6)	
H3	0.6770	0.1171	0.6373	0.055*	
C4	0.4680 (3)	0.1260 (2)	0.56719 (17)	0.0398 (5)	
H4	0.4220	0.0853	0.6259	0.048*	
C5	0.3884 (3)	0.16841 (17)	0.47115 (15)	0.0308 (4)	
C6	0.6491 (2)	0.31692 (18)	0.28856 (16)	0.0310 (4)	
C7	0.2218 (3)	0.15601 (18)	0.44770 (17)	0.0339 (5)	
C8	0.7310 (3)	−0.0243 (2)	0.15919 (17)	0.0369 (5)	
C9	0.5995 (3)	−0.0532 (2)	0.13323 (17)	0.0400 (5)	
H9	0.6068	−0.1179	0.1042	0.048*	
C10	0.4521 (3)	0.03249 (19)	0.15849 (15)	0.0330 (4)	
C11	0.2751 (3)	0.0397 (2)	0.14480 (18)	0.0418 (5)	
H11A	0.2067	0.0675	0.2017	0.063*	
H11B	0.2726	−0.0397	0.1446	0.063*	
H11C	0.2318	0.0960	0.0792	0.063*	
C12	0.9178 (3)	−0.0810 (2)	0.1495 (2)	0.0549 (7)	
H12A	0.9611	−0.0264	0.0971	0.082*	
H12B	0.9424	−0.1565	0.1292	0.082*	
H12C	0.9699	−0.0965	0.2160	0.082*	
C13	0.2400 (2)	0.34373 (19)	0.00957 (15)	0.0327 (4)	
C14	0.1089 (3)	0.3533 (2)	−0.05258 (16)	0.0361 (5)	
H14	0.1106	0.3631	−0.1252	0.043*	
C15	−0.0231 (3)	0.34544 (19)	0.01417 (16)	0.0340 (5)	
C16	−0.1946 (3)	0.3455 (3)	−0.0043 (2)	0.0491 (6)	
H16A	−0.1863	0.2693	−0.0190	0.074*	
H16B	−0.2450	0.4114	−0.0632	0.074*	
H16C	−0.2632	0.3558	0.0572	0.074*	
C17	0.4138 (3)	0.3448 (3)	−0.02341 (18)	0.0479 (6)	
H17A	0.4612	0.3677	0.0286	0.072*	
H17B	0.4089	0.4027	−0.0899	0.072*	
H17C	0.4832	0.2649	−0.0302	0.072*	
C18	−0.0812 (3)	0.5490 (2)	0.36524 (17)	0.0381 (5)	
C19	0.0209 (3)	0.6189 (2)	0.35379 (19)	0.0439 (5)	
H19	−0.0057	0.6941	0.3704	0.053*	
C20	0.1734 (3)	0.5549 (2)	0.31215 (17)	0.0375 (5)	
C21	0.3282 (3)	0.5905 (2)	0.2884 (2)	0.0546 (6)	
H21A	0.3927	0.5666	0.3516	0.082*	
H21B	0.2966	0.6774	0.2615	0.082*	
H21C	0.3950	0.5504	0.2368	0.082*	
C22	−0.2576 (3)	0.5668 (3)	0.4048 (2)	0.0580 (7)	
H22A	−0.3351	0.6281	0.3534	0.087*	
H22B	−0.2709	0.5926	0.4694	0.087*	
H22C	−0.2804	0.4913	0.4172	0.087*	
N1	0.45453 (19)	0.22688 (14)	0.38745 (12)	0.0272 (3)	
N2	0.4898 (2)	0.11200 (15)	0.19911 (13)	0.0322 (4)	
N3	0.6621 (2)	0.07452 (16)	0.19804 (14)	0.0342 (4)	
H3A	0.7201	0.1105	0.2200	0.041*	
N4	0.1920 (2)	0.33111 (15)	0.11058 (13)	0.0309 (4)	
N5	0.0297 (2)	0.33282 (16)	0.11091 (13)	0.0316 (4)	
H5	−0.0318	0.3265	0.1666	0.038*	
N6	0.1671 (2)	0.45011 (15)	0.29795 (13)	0.0330 (4)	
N7	0.0095 (2)	0.44975 (16)	0.33138 (13)	0.0343 (4)	
H7	−0.0280	0.3916	0.3308	0.041*	
O1	0.55090 (18)	0.33974 (14)	0.21281 (11)	0.0362 (3)	
O2	0.77947 (18)	0.34440 (15)	0.28302 (12)	0.0418 (4)	
O3	0.18031 (17)	0.18992 (13)	0.35129 (11)	0.0353 (3)	
O4	0.1395 (2)	0.11702 (16)	0.52179 (13)	0.0552 (5)	
O1W	0.0578 (2)	0.93763 (17)	0.67852 (14)	0.0487 (4)	
H1WA	0.073 (4)	0.989 (2)	0.6251 (17)	0.063 (9)*	
H1WB	0.006 (4)	0.898 (3)	0.661 (2)	0.071 (10)*	

### Atomic displacement parameters (Å^2^)

**Table:**

	*U*^11^	*U*^22^	*U*^33^	*U*^12^	*U*^13^	*U*^23^
Co1	0.02400 (16)	0.03763 (17)	0.02563 (15)	−0.01357 (12)	−0.00029 (10)	−0.00758 (11)
C1	0.0247 (10)	0.0335 (11)	0.0329 (10)	−0.0085 (8)	−0.0003 (8)	−0.0137 (8)
C2	0.0330 (12)	0.0478 (13)	0.0395 (12)	−0.0118 (10)	−0.0058 (9)	−0.0153 (10)
C3	0.0481 (14)	0.0498 (14)	0.0333 (12)	−0.0075 (11)	−0.0119 (10)	−0.0076 (10)
C4	0.0483 (13)	0.0369 (12)	0.0299 (11)	−0.0115 (10)	0.0013 (9)	−0.0046 (9)
C5	0.0355 (11)	0.0271 (10)	0.0296 (10)	−0.0097 (8)	0.0037 (8)	−0.0086 (8)
C6	0.0268 (11)	0.0354 (11)	0.0362 (11)	−0.0132 (9)	0.0039 (8)	−0.0153 (9)
C7	0.0353 (11)	0.0289 (10)	0.0399 (12)	−0.0137 (9)	0.0074 (9)	−0.0110 (9)
C8	0.0319 (11)	0.0424 (12)	0.0374 (11)	−0.0109 (9)	0.0036 (9)	−0.0145 (9)
C9	0.0385 (12)	0.0447 (13)	0.0433 (12)	−0.0144 (10)	0.0036 (10)	−0.0225 (10)
C10	0.0330 (11)	0.0400 (11)	0.0289 (10)	−0.0154 (9)	0.0009 (8)	−0.0092 (9)
C11	0.0355 (12)	0.0514 (14)	0.0454 (13)	−0.0200 (10)	0.0013 (10)	−0.0161 (11)
C12	0.0348 (13)	0.0628 (16)	0.0710 (17)	−0.0104 (12)	0.0068 (12)	−0.0330 (14)
C13	0.0281 (11)	0.0414 (12)	0.0303 (10)	−0.0142 (9)	0.0014 (8)	−0.0083 (9)
C14	0.0319 (11)	0.0495 (13)	0.0277 (10)	−0.0148 (10)	−0.0011 (8)	−0.0086 (9)
C15	0.0268 (11)	0.0412 (12)	0.0344 (11)	−0.0106 (9)	−0.0042 (8)	−0.0090 (9)
C16	0.0282 (12)	0.0743 (17)	0.0508 (14)	−0.0197 (12)	−0.0037 (10)	−0.0206 (12)
C17	0.0347 (12)	0.0744 (17)	0.0414 (13)	−0.0274 (12)	0.0070 (10)	−0.0151 (12)
C18	0.0337 (11)	0.0404 (12)	0.0400 (12)	−0.0073 (9)	−0.0020 (9)	−0.0155 (10)
C19	0.0463 (14)	0.0381 (12)	0.0518 (14)	−0.0129 (10)	0.0001 (11)	−0.0203 (10)
C20	0.0400 (12)	0.0408 (12)	0.0369 (11)	−0.0180 (10)	−0.0015 (9)	−0.0113 (9)
C21	0.0520 (15)	0.0570 (16)	0.0689 (17)	−0.0317 (13)	0.0074 (13)	−0.0232 (13)
C22	0.0368 (13)	0.0637 (17)	0.0790 (19)	−0.0108 (12)	0.0106 (13)	−0.0379 (15)
N1	0.0257 (8)	0.0297 (8)	0.0276 (8)	−0.0098 (7)	0.0012 (7)	−0.0090 (7)
N2	0.0254 (9)	0.0382 (9)	0.0344 (9)	−0.0105 (7)	0.0008 (7)	−0.0113 (7)
N3	0.0262 (9)	0.0398 (10)	0.0410 (10)	−0.0130 (8)	0.0003 (7)	−0.0147 (8)
N4	0.0228 (8)	0.0404 (10)	0.0309 (9)	−0.0122 (7)	−0.0008 (7)	−0.0083 (7)
N5	0.0230 (8)	0.0447 (10)	0.0288 (9)	−0.0138 (7)	0.0028 (7)	−0.0090 (7)
N6	0.0285 (9)	0.0383 (10)	0.0350 (9)	−0.0130 (7)	0.0016 (7)	−0.0117 (7)
N7	0.0284 (9)	0.0369 (10)	0.0430 (10)	−0.0132 (8)	0.0024 (7)	−0.0167 (8)
O1	0.0334 (8)	0.0495 (9)	0.0309 (7)	−0.0221 (7)	0.0011 (6)	−0.0073 (6)
O2	0.0316 (8)	0.0559 (10)	0.0486 (9)	−0.0259 (7)	0.0041 (7)	−0.0168 (7)
O3	0.0315 (8)	0.0417 (8)	0.0382 (8)	−0.0186 (6)	0.0023 (6)	−0.0108 (6)
O4	0.0571 (11)	0.0660 (11)	0.0486 (10)	−0.0381 (9)	0.0155 (8)	−0.0056 (8)
O1W	0.0543 (11)	0.0579 (11)	0.0447 (10)	−0.0330 (9)	0.0035 (8)	−0.0123 (9)

### Geometric parameters (Å, °)

**Table:**

Co1—N1	2.0407 (16)	C13—N4	1.337 (3)
Co1—N4	2.0798 (16)	C13—C14	1.389 (3)
Co1—O1	2.1453 (14)	C13—C17	1.492 (3)
Co1—O3	2.1522 (14)	C14—C15	1.366 (3)
Co1—N2	2.2336 (17)	C14—H14	0.9300
Co1—N6	2.2477 (17)	C15—N5	1.340 (3)
C1—N1	1.337 (2)	C15—C16	1.486 (3)
C1—C2	1.380 (3)	C16—H16A	0.9600
C1—C6	1.515 (3)	C16—H16B	0.9600
C2—C3	1.376 (3)	C16—H16C	0.9600
C2—H2	0.9300	C17—H17A	0.9600
C3—C4	1.390 (3)	C17—H17B	0.9600
C3—H3	0.9300	C17—H17C	0.9600
C4—C5	1.376 (3)	C18—N7	1.337 (3)
C4—H4	0.9300	C18—C19	1.367 (3)
C5—N1	1.331 (2)	C18—C22	1.491 (3)
C5—C7	1.526 (3)	C19—C20	1.398 (3)
C6—O2	1.241 (2)	C19—H19	0.9300
C6—O1	1.267 (2)	C20—N6	1.336 (3)
C7—O4	1.229 (2)	C20—C21	1.489 (3)
C7—O3	1.270 (2)	C21—H21A	0.9600
C8—N3	1.338 (3)	C21—H21B	0.9600
C8—C9	1.362 (3)	C21—H21C	0.9600
C8—C12	1.494 (3)	C22—H22A	0.9600
C9—C10	1.393 (3)	C22—H22B	0.9600
C9—H9	0.9300	C22—H22C	0.9600
C10—N2	1.339 (3)	N2—N3	1.365 (2)
C10—C11	1.488 (3)	N3—H3A	0.8600
C11—H11A	0.9600	N4—N5	1.359 (2)
C11—H11B	0.9600	N5—H5	0.8600
C11—H11C	0.9600	N6—N7	1.361 (2)
C12—H12A	0.9600	N7—H7	0.8600
C12—H12B	0.9600	O1W—H1WA	0.844 (17)
C12—H12C	0.9600	O1W—H1WB	0.828 (17)
			
N1—Co1—N4	174.52 (6)	C13—C14—H14	126.9
N1—Co1—O1	75.49 (6)	N5—C15—C14	106.49 (17)
N4—Co1—O1	109.98 (6)	N5—C15—C16	121.66 (19)
N1—Co1—O3	76.60 (6)	C14—C15—C16	131.8 (2)
N4—Co1—O3	97.92 (6)	C15—C16—H16A	109.5
O1—Co1—O3	152.00 (5)	C15—C16—H16B	109.5
N1—Co1—N2	91.87 (6)	H16A—C16—H16B	109.5
N4—Co1—N2	88.21 (6)	C15—C16—H16C	109.5
O1—Co1—N2	86.36 (6)	H16A—C16—H16C	109.5
O3—Co1—N2	92.37 (6)	H16B—C16—H16C	109.5
N1—Co1—N6	88.03 (6)	C13—C17—H17A	109.5
N4—Co1—N6	91.67 (6)	C13—C17—H17B	109.5
O1—Co1—N6	95.87 (6)	H17A—C17—H17B	109.5
O3—Co1—N6	85.33 (6)	C13—C17—H17C	109.5
N2—Co1—N6	177.66 (6)	H17A—C17—H17C	109.5
N1—C1—C2	120.37 (19)	H17B—C17—H17C	109.5
N1—C1—C6	112.56 (16)	N7—C18—C19	105.87 (19)
C2—C1—C6	127.04 (18)	N7—C18—C22	121.8 (2)
C3—C2—C1	118.3 (2)	C19—C18—C22	132.4 (2)
C3—C2—H2	120.8	C18—C19—C20	106.28 (19)
C1—C2—H2	120.8	C18—C19—H19	126.9
C2—C3—C4	120.7 (2)	C20—C19—H19	126.9
C2—C3—H3	119.6	N6—C20—C19	110.63 (19)
C4—C3—H3	119.6	N6—C20—C21	122.0 (2)
C5—C4—C3	117.9 (2)	C19—C20—C21	127.3 (2)
C5—C4—H4	121.1	C20—C21—H21A	109.5
C3—C4—H4	121.1	C20—C21—H21B	109.5
N1—C5—C4	120.88 (19)	H21A—C21—H21B	109.5
N1—C5—C7	113.15 (17)	C20—C21—H21C	109.5
C4—C5—C7	125.96 (19)	H21A—C21—H21C	109.5
O2—C6—O1	125.78 (19)	H21B—C21—H21C	109.5
O2—C6—C1	119.27 (18)	C18—C22—H22A	109.5
O1—C6—C1	114.94 (16)	C18—C22—H22B	109.5
O4—C7—O3	126.1 (2)	H22A—C22—H22B	109.5
O4—C7—C5	118.49 (19)	C18—C22—H22C	109.5
O3—C7—C5	115.42 (17)	H22A—C22—H22C	109.5
N3—C8—C9	106.06 (18)	H22B—C22—H22C	109.5
N3—C8—C12	122.1 (2)	C5—N1—C1	121.79 (17)
C9—C8—C12	131.8 (2)	C5—N1—Co1	118.58 (13)
C8—C9—C10	106.82 (19)	C1—N1—Co1	119.63 (13)
C8—C9—H9	126.6	C10—N2—N3	104.33 (16)
C10—C9—H9	126.6	C10—N2—Co1	133.05 (14)
N2—C10—C9	110.16 (18)	N3—N2—Co1	122.35 (12)
N2—C10—C11	122.52 (19)	C8—N3—N2	112.62 (16)
C9—C10—C11	127.32 (19)	C8—N3—H3A	123.7
C10—C11—H11A	109.5	N2—N3—H3A	123.7
C10—C11—H11B	109.5	C13—N4—N5	104.78 (15)
H11A—C11—H11B	109.5	C13—N4—Co1	130.69 (13)
C10—C11—H11C	109.5	N5—N4—Co1	123.41 (12)
H11A—C11—H11C	109.5	C15—N5—N4	112.05 (16)
H11B—C11—H11C	109.5	C15—N5—H5	124.0
C8—C12—H12A	109.5	N4—N5—H5	124.0
C8—C12—H12B	109.5	C20—N6—N7	104.03 (16)
H12A—C12—H12B	109.5	C20—N6—Co1	140.31 (14)
C8—C12—H12C	109.5	N7—N6—Co1	115.57 (12)
H12A—C12—H12C	109.5	C18—N7—N6	113.19 (17)
H12B—C12—H12C	109.5	C18—N7—H7	123.4
N4—C13—C14	110.39 (17)	N6—N7—H7	123.4
N4—C13—C17	121.13 (18)	C6—O1—Co1	117.38 (12)
C14—C13—C17	128.48 (19)	C7—O3—Co1	115.59 (12)
C15—C14—C13	106.29 (18)	H1WA—O1W—H1WB	110 (3)
C15—C14—H14	126.9		

### Hydrogen-bond geometry (Å, °)

**Table:**

*D*—H···*A*	*D*—H	H···*A*	*D*···*A*	*D*—H···*A*
N3—H3A···O1W^i^	0.86	2.22	2.926 (2)	139
N3—H3A···O1	0.86	2.61	3.048 (2)	113
N5—H5···O2^ii^	0.86	2.10	2.945 (2)	168
N7—H7···O2^ii^	0.86	2.08	2.838 (2)	146
N7—H7···O3	0.86	2.42	2.906 (2)	116
O1W—H1WA···O4^iii^	0.84 (2)	1.97 (2)	2.797 (2)	168 (3)
O1W—H1WB···O3^iv^	0.83 (2)	2.20 (2)	3.009 (2)	164 (3)

Symmetry codes: (i) −*x*+1, −*y*+1, −*z*+1; (ii) *x*−1, *y*, *z*; (iii) *x*, *y*+1, *z*; (iv) −*x*, −*y*+1, −*z*+1.
